# Evaluation of scientific outcomes of TDR-supported clinical research and development fellows in low- and middle-income countries: a bibliometric analysis

**DOI:** 10.1186/s40249-026-01474-1

**Published:** 2026-07-01

**Authors:** Mahnaz Vahedi, Prabin Dahal, Elham Jaberi, Dawit Getachew Assefa, Wastiq Maula, Nuria Casamitjana, Pascal Launois

**Affiliations:** 1 World Health Organization, The Special Programme for Research and Training in Tropical Diseases (WHO/TDR), Geneva, Switzerland; 2 IS Global, University of Barcelona, Barcelona, Spain; 3 Infectious Diseases Data Observatory (IDDO), Oxford, UK; 4 Centre for Tropical Medicine and Global Health, Nuffield Department of Medicine, University of Oxford, Oxford, UK; 5 Department of Nursing, College of Health Science and Medicine, Dilla University, Dilla, Ethiopia; 6 Universitat Gadjah Mada, Yogyakarta, Indonesia

**Keywords:** Clinical research fellowship, Research capacity strengthening, Bibliometric analysis, Low- and middle-income countries

## Abstract

**Background:**

The Special Programme for Research and Training in Tropical Diseases (TDR), co-sponsored by World Health Organization, United Nations Children’s Fund, United Nations Development Programme, and the World Bank, launched the Clinical Research and Development Fellowship (CRDF) in 1999 to strengthen clinical research capacity in low- and middle-income countries (LMICs). Despite sustained investment, evidence on the longer-term scientific outcomes of such programmes remains limited. This study evaluated the scientific outcomes of the CRDF programme (1999–2021) using bibliometric analysis of fellows’ peer-reviewed publications before and after their fellowship.

**Methods:**

A retrospective bibliometric analysis was conducted on peer-reviewed publications authored by CRDF fellows from 1999 through 2021. Publication data were retrieved from Open Researcher and Contributor ID profiles and verified using PubMed, Google search, and Google scholar. A retrospective bibliometric analysis was conducted using descriptive and comparative statistical methods to evaluate publication productivity, authorship position, journal quality metrics, collaboration patterns, disease focus, and research themes before and after fellowship participation. Analyses were conducted using R statistical software. Scientific outcome of the fellowship was judged based on the evolution of collaboration network, journal metrics, and seniority in authorship.

**Results:**

Among 128 fellows, 76 had verifiable publications, contributing a total of 1821 peer-reviewed articles. Publications were predominantly produced after fellowship participation (1309; 71.9%), compared with 512 pre-fellowship publications. Median publications per fellow increased from 5 [interquartile range (IQR): 2–10] before the fellowship to 14 (IQR: 8–28). The proportion of first-author publications declined (30.7–19.2%), while last-author publications increased (8.0–11.0%), suggesting progression toward senior research roles. Journal quality improved, with publications in Q1 journals increasing from 66.6 to 71.3%, and the median impact factor rising from 2.8 (IQR: 1.9–3.9) to 3.4 (IQR: 2.6–4.9). North–South collaborations increased substantially after the fellowship, while South–South collaborations grew modestly.

**Conclusions:**

Participation in the CRDF programme was associated with a visible increase in research productivity, publication quality, and international collaboration among fellows. Research output aligned closely with infectious diseases of poverty and evolving global health priorities. While the collaboration network expanded, leadership as gauged by the evolution of authorship position remained constrained, highlighting the need for continued investment in research leadership development. Bibliometric analysis offers a useful approach for examining longer-term scientific outcomes of capacity-strengthening initiatives, while recognizing its limitations in capturing broader policy and societal impacts.

**Supplementary Information:**

The online version contains supplementary material available at 10.1186/s40249-026-01474-1.

## Background

Low- and middle-income countries (LMICs) bear a disproportionate burden of poverty-related infectious diseases. Insufficient resources and research infrastructure limit the production and application of contextually appropriate evidence [1]. Strengthening clinical research capacity is essential for improving study design, implementation, and translation of research into policy and practice for diseases such as malaria, tuberculosis (TB), and neglected tropical diseases (NTDs), and for advancing Universal Health Coverage [2] and the Sustainable Development Goals [3]. The Special Programme for Research and Training in Tropical Diseases (TDR) was established in 1975 to support research and training focused on infectious diseases of poverty in LMICs and it was co-sponsored by United Nations Children’s Fund, United Nations Development Programme, the World Bank, and the World Health Organization (WHO) [4].

As part of its long-standing commitment to research capacity strengthening, the TDR launched the Clinical Research and Development Fellowship (CRDF) programme in 1999, initially in collaboration with GlaxoSmithKline (GSK) Biologicals [5]. Subsequently, support from the Bill & Melinda Gates Foundation, together with collaborations involving pharmaceutical companies, product development partnerships (PDPs), universities, and public research institutions, enabled the programme to expand [6]. From 2014, collaboration with the European & Developing Countries Clinical Trials Partnership (EDCTP) expanded opportunities for fellows from sub-Saharan Africa while maintaining a global scope [7]. The CRDF programme concluded in 2021, after which the TDR transitioned its fellowship portfolio toward Clinical Research Leadership fellowship which is a one-year competitive fellowship placement [8].

This one-year placement offered practical clinical research training primarily at pharmaceutical companies, PDPs, and research centers located in high-income countries. During the placement, fellows were expected to acquire hands-on expertise in areas including leadership, clinical trial processes, data management, quality control, and scientific writing. Upon completion of the fellowship, re-entry funding was provided to support their return to their home institutions. Alumni have contributed to research addressing major infectious diseases of poverty and related health system challenges, and a 2020 COVID-19 survey indicated that most fellows remained actively engaged in clinical research, commonly in trial management and coordination roles [7].

However,  evidence from systematic evaluations of the long-term scientific outcomes of such initiatives has remained limited, despite continuous global investment in research capacity strengthening [9–12]. Specifically, there is inadequate empirical data on how clinical research training affects  scientific productivity, collaboration dynamics, publication standards, and career advancement toward leadership positions among LMIC-based researchers. Documenting these outcomes is essential for determining whether training programs support not only individual competency building but also durable research environments capable of sustaining high-caliber investigations into poverty-related infectious diseases [13].

Because research outcomes and collaborations are shaped by many contextual, organisational, and career-related factors, evaluating the effectiveness of a training programme is inherently complex.  A contribution-based evaluation thus provides a practical methodology for examining how training initiatives generate value within intricate research systems [14, 15]. Bibliometric analysis provides a structured approach to evaluating scientific output using metrics such as publication counts, journal characteristics, authorship position, research themes, and patterns of collaboration and co-authorship [16]. Although bibliometric approaches have been employed to evaluate global health research consortia and training schemes [17], evidence remains limited for clinical research fellowships administered by multilateral organizations like WHO/TDR that concentrate on poverty-related infectious diseases.

This investigation fills this knowledge gap through bibliometric analysis of peer-reviewed articles produced by CRDF fellows from 1999 through 2021. Bibliometric analysis was selected because the objective of this study was to evaluate research productivity, authorship roles, journal characteristics, and collaboration patterns rather than to synthesise clinical evidence addressing a single research question. Unlike systematic reviews or meta-analyses, which aim to pool results from studies with comparable designs and outcomes, bibliometric methods are specifically suited to examining structural and output-related dimensions of scientific activity across heterogeneous research domains.

Specifically, bibliometric analysis enables examination of publication volume, journal quality, authorship position (as a proxy for research leadership), collaboration patterns (including North–South and South–South partnerships), and thematic focus on infectious diseases of poverty before and after fellowship participation; all of these remain important  for evaluating the long-term scientific outcomes of the fellowship. By aligning bibliometric indicators with programme objectives, this study provides evidence to inform future investments in clinical research training and capacity strengthening in LMICs, while acknowledging the limitations of bibliometric indicators in capturing broader policy and societal impacts.

## Methods

### Study design and setting

We conducted a retrospective bibliometric analysis of peer-reviewed publications authored by CRDF fellows. Publications produced before and after fellowship participation were examined to assess changes in research productivity, collaboration patterns, authorship roles, and thematic focus. For each fellow, the publication cut-off was defined by the fellowship start date to compare pre- and post-fellowship output. Data extraction and analysis were conducted between March and August 2025.

Bibliometric analysis was used as a contribution-oriented evaluation approach [15], recognizing that scientific outcomes reflect the interaction of training, institutional context, and career trajectories rather than the effect of a single intervention.

### Data sources and identification of publications

All peer-reviewed publications authored by fellows who participated in the CRDF programme from its inception in 1999 through the final cohort in 2021 were retrieved from Open Researcher and Contributor ID (ORCID) profiles and cross-verified using PubMed, Google Scholar, Google Search, and journal websites to confirm authorship and bibliographic information [18]. ORCID profiles were reviewed to confirm fellows’ association with TDR. However, ORCID registration was not mandatory for CRDF participation. ORCID identifiers were used where available to improve author disambiguation and reduce misclassification across databases. For fellows without an ORCID identifier or when records appeared incomplete, supplementary searches were conducted using combinations of name, institutional affiliation, country, and other identifying information. Publications were cross-verified using PubMed and journal websites to confirm authorship.

### Inclusion and exclusion criteria

Eligible outputs were peer-reviewed research articles published in any language. Non-peer-reviewed materials (conference abstracts, editorials, preprints, books, commentaries, reports, and letters) were excluded. Publication lists were cross-checked by verifying article titles and digital object identifiers (DOIs) in PubMed. For journals not indexed in PubMed, article titles were searched in Google scholar to confirm bibliographic details. Publications appearing in the records of multiple fellows were counted separately for each fellow.

Publications were classified according to publication year relative to the fellowship start date. We acknowledge that research initiation, data collection, and manuscript preparation may precede publication by months or years. Conversely, revisions, analyses, or manuscript development may have benefited from knowledge and collaborations gained during the fellowship even if the study was initiated earlier.

Submission and study initiation dates were not consistently available across journals and were therefore not used. Publication year was selected as the most standardized and reproducible bibliometric time indicator.

### Author affiliation and geographic classification

Author affiliations were identified using information from PubMed, Google searches, or full-text articles. Institutional affiliation and country were recorded for each publication. Fellows’ countries of affiliation at the time of fellowship award were mapped to WHO regions and World Bank income classifications. WHO regions included Africa, Americas, South-East Asia, Europe, Eastern Mediterranean, and Western Pacific [19]. World Bank income categories (low, lower-middle, upper-middle, and high income) were defined using Gross National Income (GNI) per capita (Atlas method) [20, 21].

### Database development and quality control

Publication metadata were entered into a structured Excel database using predefined variables, with each row representing a unique peer-reviewed publication. Data retrieval and extraction were independently conducted by three researchers (MV, EJ, WM) to minimize bias and enhance reliability.

### Bibliometric analysis framework

This study applied two complementary bibliometric approaches: performance analysis and collaboration assessment, consistent with established bibliometric methodology.

Performance analysis was used to evaluate scientific productivity, publication characteristics, and indicators of academic leadership. Metrics included: publication counts (overall and median per fellow), authorship position (first, middle, last author), journal quality indicators (SCImago quartile ranking, impact factor, Journal Citation Indicator, SCImago Journal Rank, journal H-index), open-access status, research domain and disease area classification, and study design classification. These metrics were selected to align with the programme’s objectives of strengthening research productivity, leadership capacity, and dissemination in high-quality journals.

### Publication characteristics

The total number of publications per fellow was summarized and stratified by WHO region and gender where relevant.

### Journal-level metrics

Journal metrics were obtained from SCImago [22], including SCImago Journal Rank (SJR), quartile ranking (Q1–Q4), and journal H-index. Impact Factor (IF) and Journal Citation Indicator (JCI) were retrieved from Clarivate Journal Citation Reports [23]. Open-access status was determined using the Directory of Open Access Journals (DOAJ) [24], classifying journals as fully open access or non-fully open access (hybrid or subscription-based). Data on the journal metrics are updated annually, metric values were extracted corresponding to the publication year of each article whenever available. When year-specific metrics were unavailable, the closest available annual metric was used. Publications in journals not indexed in SCIE/Web of Science or SCImago were retained in the bibliometric dataset; however, journal-specific indicators such as Impact Factor, Journal Citation Indicator, quartile ranking, and SCImago Journal Rank were treated as missing because these metrics were not available.

### Collaboration and network assessment

Collaboration was assessed using institutional affiliations [25]. Publications were classified as national (single-country) or international (multi-country). International collaborations were further categorized as North–South, South–South, or North–North using World Bank income classifications, where “North” refers to high-income countries and “South” to low- and middle-income countries [20].

Advanced science mapping techniques such as co-citation analysis, co-word analysis, or bibliographic coupling were not conducted because the objective was not to map intellectual structures or research fronts, but rather to evaluate temporal changes in output and collaboration patterns.

### Disease area, research area, and study design

#### Disease area

Publications were classified by disease area, including neglected tropical diseases, HIV, tuberculosis, malaria, antimicrobial resistance, COVID-19, other infectious diseases, and selected non-communicable conditions, in line with TDR strategies and CRDF priorities [4, 26, 27].

Publications were classified according to their primary research area and study design. Research areas included:

Clinical trials: studies evaluating the safety and efficacy of interventions in human participants. International Council for Harmonization of Technical Requirements for Pharmaceuticals for Human Use (ICH). E6(R2): Guideline for Good Clinical Practice. 2016 [1, 26, 28, 29];

Clinical research: other patient-centered studies, including observational studies, diagnostic studies, and treatment evaluations not formally classified as clinical trials [28];

Health systems: studies examining health service delivery, policy, management, and system-level interventions [30];

Basic research: laboratory or preclinical studies aimed at understanding biological mechanisms, drug discovery, or experimental models [31];

These research areas were selected to reflect the main types of research supported by the CRDF programme and to align with TDR priorities [8].

Study designs were categorized as randomized or non-randomized (comparative) clinical trials, cross-sectional studies, other observational studies, cohort/longitudinal studies, evidence syntheses, experimental/laboratory studies, and modelling studies. Publications were additionally classified into two thematic focus areas: (1) drugs, vaccines, and diagnostics, and (2) maternal and child health, reflecting priority global health domains [26, 27].

### Career advancement and leadership indicators

Authorship position was used as a proxy for career progression and leadership, examining shifts from first or co-authorship toward senior (last) authorship roles in national and international publications.

### Comparison of characteristics before and after fellowship

Publications were classified as pre- or post-fellowship based on publication year relative to the fellowship start date. Publications published during the fellowship year and thereafter were considered post-fellowship. Selected characteristics were compared descriptively between periods.

### Statistical analysis

The unit of analysis was a publication. Descriptive bibliometric analyses were conducted to summarize publication productivity, authorship position, collaboration patterns, journal characteristics, research areas, and study designs. Bibliometric indicators were summarized using frequencies and percentages for categorical variables and medians with interquartile ranges (IQRs) and ranges for continuous variables. Comparative descriptive analyses were performed between pre- and post-fellowship publication periods. Analyses were additionally stratified by WHO region and gender where relevant.

Data management, harmonisation, and bibliometric analyses were conducted in R (RStudio environment) version 4.4.1. The workflow included scripted procedures for data cleaning, verification, and derivation of bibliometric indicators, enabling reproducibility and reducing manual processing errors. Analyses used R packages for data import, wrangling, and validation, primarily from the tidyverse suite (e.g., readr/readxl, dplyr, tidyr, stringr, janitor), as well as ggplot2 for visualisation and gtsummary for tabulation and reporting. Collaboration classifications (national/international; North–South/South–South/North–North) were derived from author affiliation countries and mapped to WHO regions and World Bank income categories using rule-based scripts. The dataset and R code are publicly available on GitHub (https://github.com/dawitgetachewa/WHO-TDR-CRDF-Bibliometric).

### Ethical considerations

This study used publicly available publication data and did not involve human or animal subjects. Ethical approval was therefore not required. ORCID identifiers were used solely for public data retrieval, and no private or sensitive information was accessed.

## Results

### Description of the fellows

**Fig. 1:**
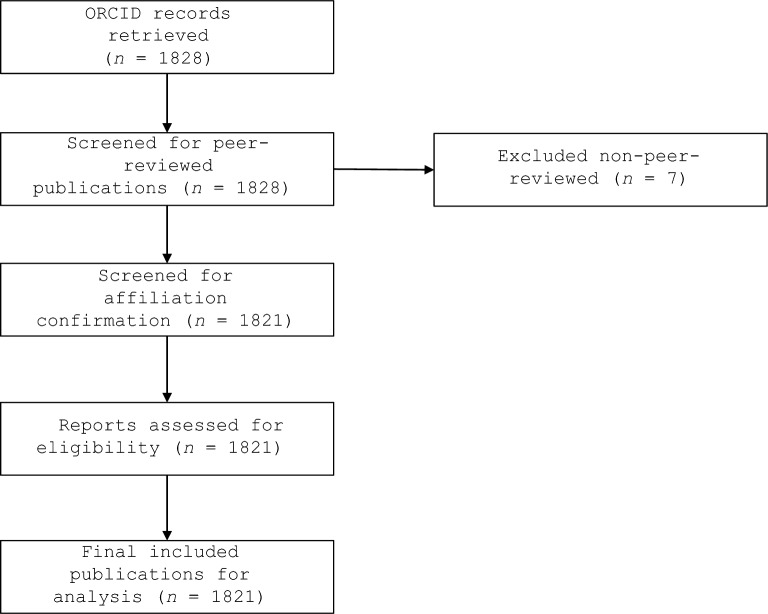
Flow chart of inclusion

Between 1999 and 2021, a total of 128 fellows enrolled in the programme: 27 (21%) during the 2009–2013 period, 48 (38%) during 2014–2018, and 53 (41.4%) between 2019 and 2021 (Supplemental Table 1). Of these, 76 (59.4%) had verifiable peer-reviewed publications linked to their ORCID records and were included in this bibliometric analysis. After exclusion of non–peer-reviewed outputs and duplicate records, these fellows contributed a total of 1821 eligible publications (Fig. 1). The demographic and regional characteristics of the fellows are summarized in Table 1.

**Table 1:** Demographic characteristics and training partner organization (TPO) distribution of WHO/TDR Clinical Research and Development Fellowship participants included in the bibliometric analysis, by WHO region, 1999–2021 (*N* = 76)

Characteristic	African Region (*N* = 63)	American Region (*N* = 6)	South-East Asian Region (*N* = 5)	Western Pacific Region (*N* = 2)	Overall (*N* = 76)
Time period	1999–2021	2012–2021	2016–2020	2011–2014	1999–2021
Number of countries	22	4	3	2	31
Fellow gender					
Men	50 (79.4%)	3 (50.0%)	4 (80.0%)	2 (100%)	59 (77.6%)
Women	13 (20.6%)	3 (50.0%)	1 (20.0%)	–	17 (22.4%)
TPO WHO region					
African Region	2 (3.2%)	–	–	–	2 (2.6%)
European Region	51 (81.0%)	4 (66.7%)	2 (40.0%)	–	57 (75.0%)
American Region	5 (7.9%)	2 (33.3%)	1 (20.0%)	–	8 (10.5%)
Western Pacific Region	4 (6.4%)	–	1 (20.0%)	1 (50.0%)	6 (7.9%)
Eastern Mediterranean Region	–	–	–	–	–
South-East Asia Region	–	–	–	–	–
Multiple regions	1 (1.6%)	–	1 (20.0%)	1 (50.0%)	3 (3.9%)

Values are presented as frequency (%)

*IQR *interquartile range, *TPO* Training Partner Organization, *WHO* World Health Organization, *TDR* Special Programme for Research and Training in Tropical Diseases

Among the 76 fellows, 17 (22.4%) were women and 59 (77.6%) were men. The median age at the start of the fellowship was 36 years [interquartile range (IQR): 34–39 years; range: 26–49 years]. Fellows originated from 31 countries, with the majority from the WHO African Region (AFR) (63; 82.9%), followed by the Region of the Americas (AMR) (6; 7.9%), the South-East Asia Region (SEAR) (5; 6.6%), and the Western Pacific Region (WPR) (2; 2.6%). According to World Bank income classification, 24 fellows (31.6%) were from low-income countries, 42 (55.3%) from LMICs, and 10 (13.2%) from upper-middle-income countries.

### Scientific research productivity

A total of 1821 publications were identified for 76 of the 128 fellows (59.4%). Of these publications, 1494 (82.0%) were authored by 59 men and 327 (8.0%) by 17 women. By WHO region, 1446 publications (79.4%) were attributed to fellows from AFR, 228 (12.5%) to AMR, 105 (5.8%) to SEAR, and 42 (2.3%) to WPR.

The median number of publications per fellow was 16 (IQR: 7–30; range: 1–135) (Table 2). Regional summaries of publication volume, authorship position, and timing relative to the fellowship are presented in Table 2.

**Table 2:** Research productivity, authorship position, and publication timing among WHO/TDR CRDF fellows by WHO region, 1999–2021 (76 fellows; 1821 publications)

Characteristic	African Region (*N* = 63)	American Region (*N* = 6)	South-East Asian Region (*N* = 5)	Western Pacific Region (*N* = 2)	Overall (*N* = 76)
Time period	1999–2021	2012–2021	2016–2020	2011–2014	1999–2021
Total number of publications	1446	228	105	42	1821
Median/fellow (IQR; range)	14 (7–24; 1–135)	34 (25–39; 3–95)	17 (15–32; 7–34)	21 (25–28; 8–34)	16 (7–30; 1–135)
Authorship position (*N* = 1821 publications)					
First author	309 (21.4%)	60 (26.3%)	20 (19.0%)	19 (45.2%)	408 (22.4%)
Middle author	1003 (69.4%)	128 (56.1%)	76 (72.4%)	21 (50.0%)	1228 (67.4%)
Last author	134 (9.3%)	40 (17.5%)	9 (8.6%)	2 (4.8%)	185 (10.2%)
Publication timing					
Before fellowship	385 (26.7%)	105 (46.1%)	22 (21.0%)	0 (0%)	512 (28.1%)
After fellowship	1061 (73.3%)	123 (53.9%)	83 (79.0%)	42 (100%)	1309 (71.9%)

Values are presented as frequency (%) unless otherwise indicated

*IQR* interquartile range, *WHO* World Health Organization, *TDR* Special Programme for Research and Training in Tropical Diseases, *CRDF* Clinical Research and Development Fellowship

### Journal metrics

Bibliometric characteristics of the journals are presented in Table 3. Publications were distributed across journal quartiles before and after the fellowship periods, with Q1-ranked journals accounting for 66.6% of publications before the fellowship and 71.3% after the fellowship.

**Table 3:** Comparison of journal quality indicators and open-access characteristics of publications authored by WHO/TDR Clinical Research and Development Fellowship fellows before and after fellowship participation, 1999–2021 (*N* = 1821 publications)

Characteristic	Before fellowship publication (*N* = 512)	After fellowship publication (*N* = 1309)	Overall publication (*N* = 1821)
Journal quartiles			
Q1	341 (66.6%)	933 (71.3%)	1274 (70.0%)
Q2	65 (12.7%)	180 (13.8%)	245 (13.5%)
Q3	68 (13.3%)	98 (7.5%)	166 (9.1%)
Q4	8 (1.6%)	31 (2.4%)	39 (2.1%)
Not graded	30 (5.9%)	67 (5.1%)	97 (5.3%)
IF			
Complete records	479	1215	1694 (93.0%)
Missing	33 (6.4%)	94 (7.2%)	127 (7.0%)
Median IF (IQR; range)	2.8 (1.9–3.9; 0.1–74.7)	3.4 (2.6–4.9; 0.1–176.0)	3.2 (2.4–4.6; 0.1–176.1)
Publications with IF > 10	25 (4.9%)	149 (11.4%)	174 (9.6%)
Journal H-index			
Complete records	496	1276	1772 (97.3%)
Missing	16 (3.1%)	33 (2.5%)	49 (2.7%)
Median H-index (IQR; range)	133 (76–233; 0–1442)	134 (74.8–231; 0–1231)	133 (75–233; 0–1442)
JCI			
Complete records	459	1204	1663 (91.3%)
Missing	53 (10.4%)	105 (8.0%)	158 (8.7%)
Median JCI (IQR; range)	0.91 (0.60–1.19; 0.01–24.7)	0.93 (0.71–1.32; 0.05–25.3)	0.92 (0.68–1.30; 0.01–25.3)
SCISJR score			
Complete records	482	1244	1726 (94.8%)
Missing	30 (5.9%)	65 (5.0%)	95 (5.2%)
Median SJR score (IQR; range)	1.33 (0.72–2.10; 0.10–18.3)	1.15 (0.83–1.99; 0.12–26.0)	1.21 (0.82–2.01; 0.10–26.0)
Open-access information			
Open access	238 (46.5%)	814 (62.2%)	1052 (57.8%)
Hybrid access	132 (25.8%)	263 (20.1%)	395 (21.7%)
Not open access	93 (18.2%)	157 (12.0%)	250 (13.7%)
No data	49 (9.6%)	75 (5.7%)	124 (6.8%)

Values are presented as frequency (%) unless otherwise indicated

*IQR* interquartile range, *IF* impact factor, *JCI* Journal Citation Indicator, *SJR *SCImago Journal Rank

Journal impact factor data were available for 1694 publications (93%). The median impact factor was 2.8 (IQR: 1.9–3.9; range: 0.1–74.7) before the fellowship and 3.4 (IQR: 2.6–4.9; range: 0.1–176.0). Publications in journals with an impact factor greater than 10 accounted for 4.9% before and 11.4% after the fellowship (Table 3).

Journal H-index, Journal Citation Indicator (JCI), and SCImago Journal Rank (SJR) scores showed comparable distributions before and after the fellowship period. Detailed journal metrics are provided in Table 3, with additional visual summaries presented in Supplemental Fig. 1.

### Leadership roles and academic seniority

Across all 1821 publications, fellows were listed as first authors in 408 publications (22.4%), co-authors in 1228 publications (67.4%), and last authors in 185 publications (10.2%). Regional distributions of authorship position are shown in Table 1.

**Fig. 2:**
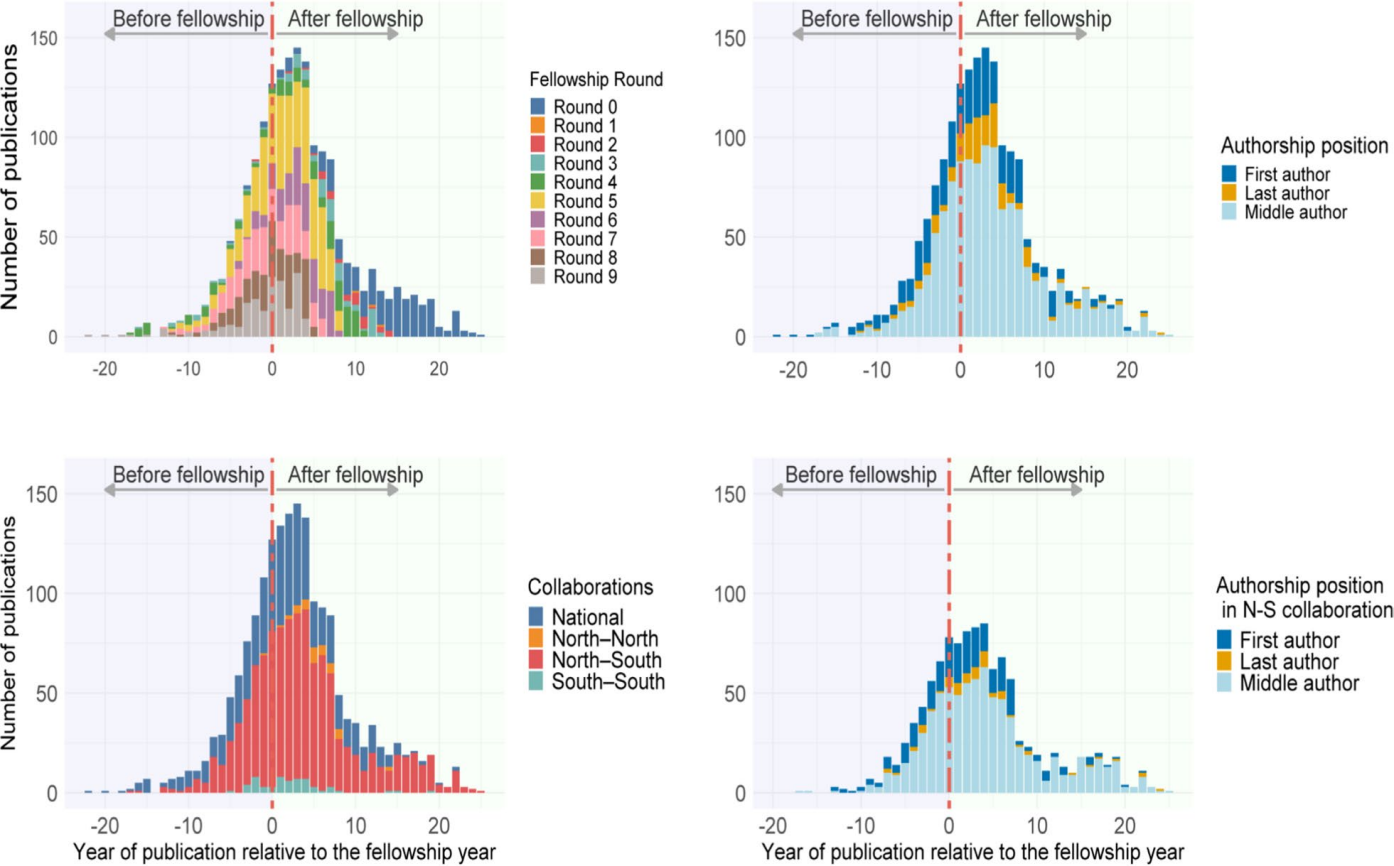
Distribution of publications before and after fellowships. Round 0 describes the period before 2010, and Round 1 describes 2010 and then yearly afterwards

When stratified by time relative to the fellowship, 19.2% (251/1309) of post-fellowship publications listed fellows as a first author, 69.8% (914/1309) as co-authors, and 11.0% (144/1309) as the last author. Prior to the fellowship, the corresponding proportions were 30.7% (157/512), 61.3% (314/512), and 8.0% (41/512), respectively. Authorship distributions by fellowship round (year) are presented in Fig. 2.

Among the 1103 publications involving North–South collaboration, 280 were published prior to the fellowship. In these, fellows were first authors in 29.3% (82/280), co-authors in 66.8% (187/280), and last authors in 3.9% (11/280). Of the 823 North–South collaborative publications after the fellowship, fellows were first authors in 20.7% (170/823), co-authors in 73.6% (606/823), and last authors in 5.7% (47/823) (Fig. 2D).

### Research areas and study design

**Fig. 3:**
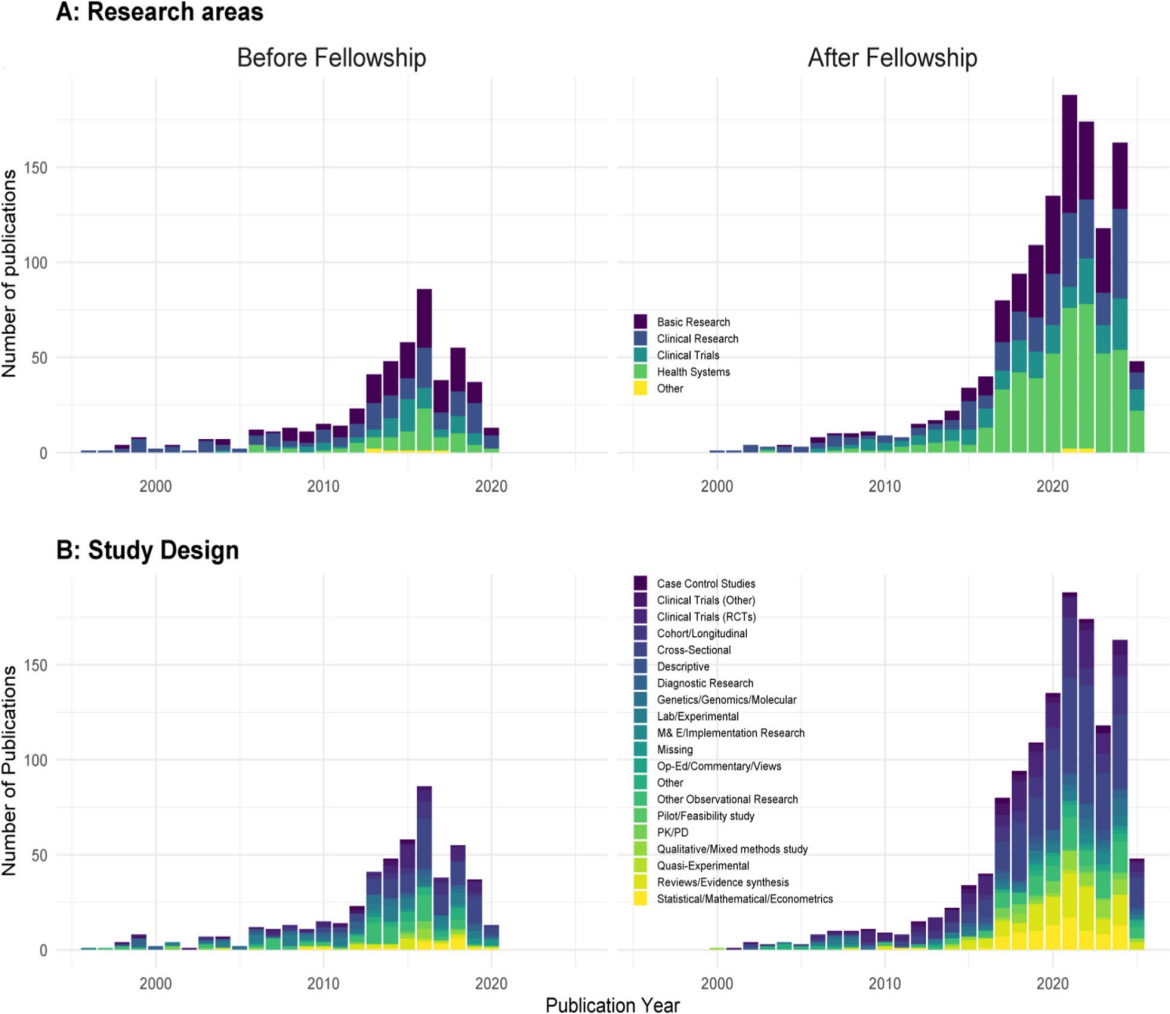
Research areas and study design across time-period. *PK/PD* pharmacokinetics/pharmacodynamics, *Op-Ed* opinion and editorials, *RCTs* randomized controlled trials, *M & E* Monitoring and evaluation

Of the 1821 publications, 568 (31.2%) addressed health systems research, 514 (28.2%) basic research, 453 (24.9%) clinical research, 276 (15.2%) clinical trials, and 10 (0.5%) other research areas (Fig. 3A).

Regarding study design, 443 publications (24.3%) were cross-sectional studies, 202 (11.1%) randomized controlled trials, 162 (8.8%) cohort or longitudinal studies, 151 (8.2%) evidence syntheses (reviews or meta-analyses), 130 (7.0%) other observational studies, 109 (5.8%) modelling studies, and 107 (5.9%) experimental or laboratory studies. Distributions before and after the fellowship are shown in Fig. 3B.

### Collaboration and collaborator network

**Fig. 4:**
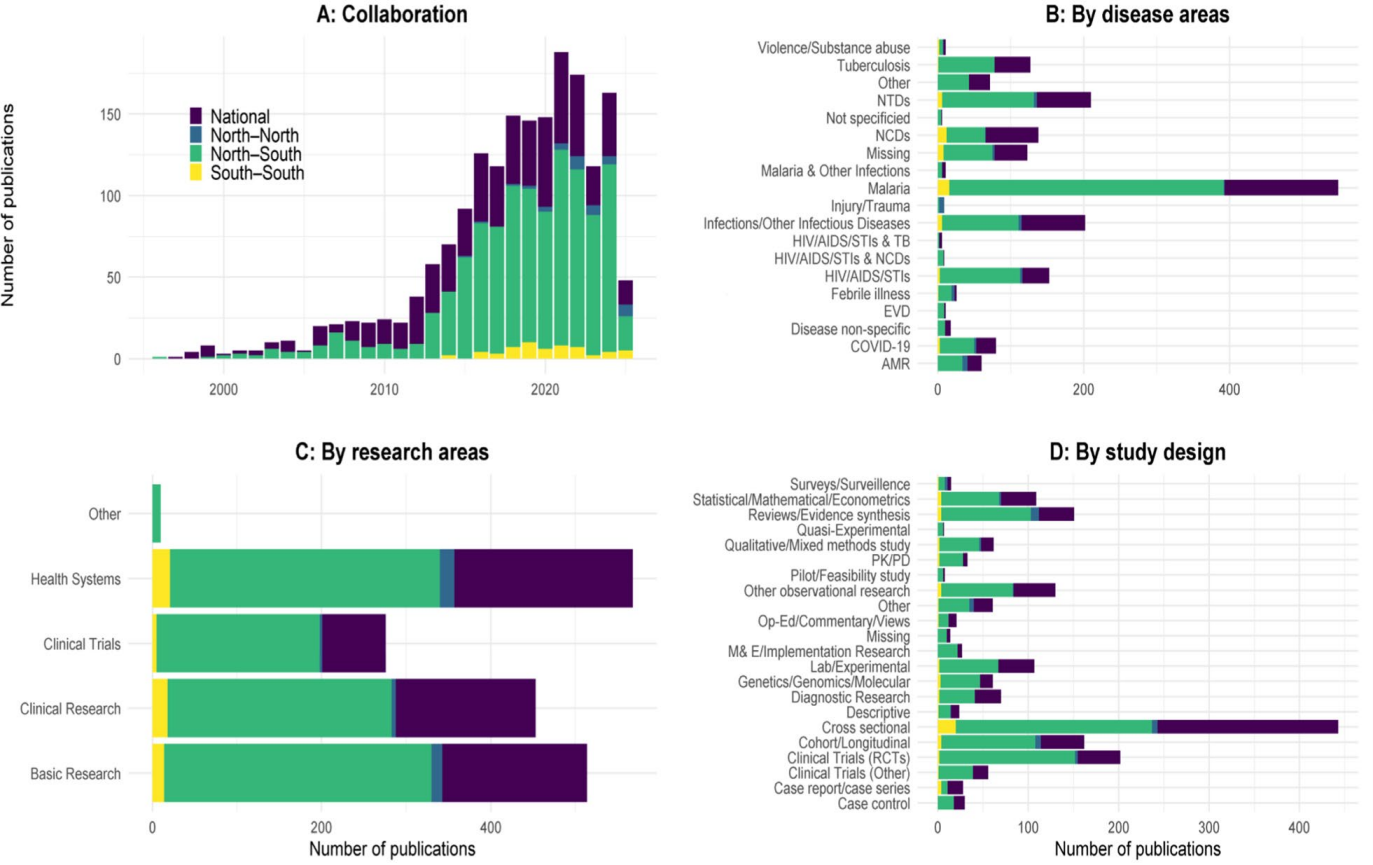
Collaborations by disease themes and research areas. *AMR* antimicrobial resistance, *EVD* Ebola virus disease, *NCDs* non-communicable diseases, *NTDs* neglected tropical diseases, *PK/PD* pharmacokinetics/pharmacodynamics, *TB* tuberculosis, *STIs* sexually transmitted infections

Collaboration patterns included North–South collaboration in 1103 publications (60.6%), national collaboration in 622 publications (34.2%), South–South collaboration in 58 publications (3.2%), and North–North collaboration in 38 publications (2.1%). Of the North–North collaborations, 37 occurred after the start of the fellowship, including publications produced during fellowship placements in high-income settings, and one involved a fellow who subsequently relocated to a high-income country for doctoral training. Further distributions of collaboration types by disease area and research domain are presented in Fig. 4.

### Contributions to institutional capacity strengthening in the global south

**Fig. 5:**
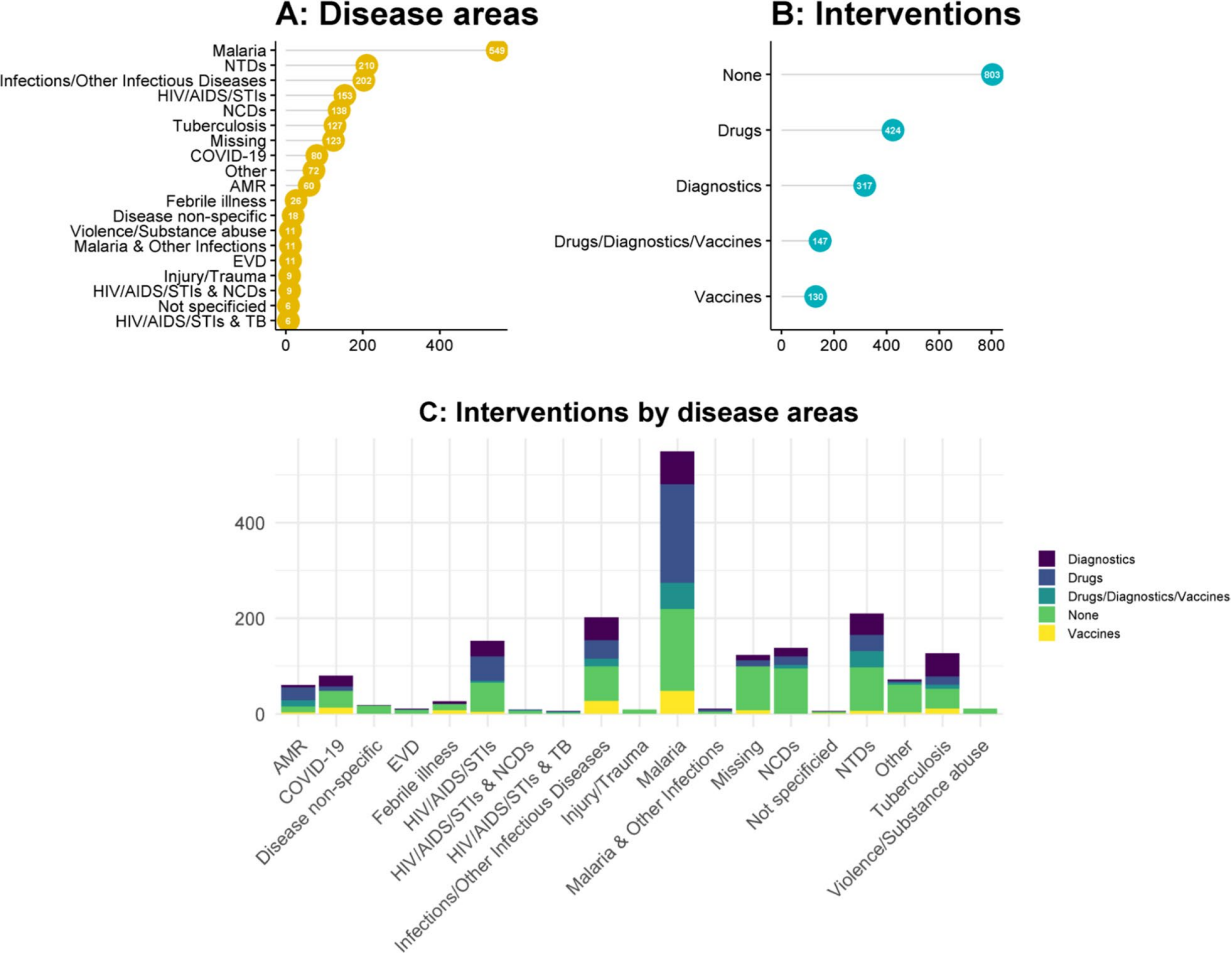
Interventions evaluated by disease areas. *HIV/AIDS* human immunodeficiency virus/acquired immunodeficiency syndrome, *AMR* Antimicrobial resistance, *EVD* Ebola Virus Disease, *NCDs* non-communicable diseases, *NTDs* Neglected tropical diseases, *TB* Tuberculosis, *STIs* Sexually transmitted infections

When stratified by disease areas, 549 publications (30.0%) addressed malaria, 210 (11.5%) NTDs, 153 (8.4%) HIV/AIDS or sexually transmitted infections, 202 (11.1%) other infectious diseases, 138 (7.6%) non-communicable diseases (NCDs), 127 (6.9%) tuberculosis, 60 (3.3%) antimicrobial resistance, and 80 (4.4%) COVID-19. Additional indication areas included injury or trauma (*N* = 9), violence or substance abuse (*N* = 11), and febrile illness (*N* = 26) (Figs. 4B and 5A). Multiple disease presentations were reported in 26 studies, and these included: HIV/AIDS/STIs and NCDs (*N* = 9), HIV/AIDS/STIs and tuberculosis (*N* = 6), and malaria and other infections (*N* = 11).

With respect to intervention focus, 424 publications (23.3%) reported research on drugs or therapeutics, 317 (17.4%) on diagnostics, 130 (7.2%) on vaccines, while 147 (8.1%) did not clearly distinguish between these categories (Fig. 5B). 457 (25.1%) of the studies were in maternal and child health areas. While infectious diseases such as malaria/HIV/TB largely dominate the research landscape, there has been a gradual increase in research on more timely and topical areas such as AMR (*n* = 60), NTDs (*n* = 210) and NCDs (*n* = 138) (Supplemental Fig. 2). Further comparison of these distributions before and after the fellowships is presented in Supplemental Fig. 3.

### Open-access information and frequently published journals

The 1821 publications appeared across 484 journals. Of these, 1052 publications (57.8%) were published in open-access journals, 395 (21.7%) in hybrid-access journals, and 250 (13.7%) in non–open-access journals; open-access status was unavailable for 124 publications (6.8%) (Table 3).

The ten journals with the highest number of publications were *PLOS One* (*N* = 142; 7.8%), *Malaria Journal* (*N* = 119; 6.5%), *PLOS Neglected Tropical Diseases* (*N* = 58; 3.1%), *The American Journal of Tropical Medicine and Hygiene* (*N* = 57; 3.1%), *Clinical Infectious Diseases* (*N* = 52; 2.8%), *BMC Infectious Diseases* (*N* = 42; 2.3%), *The Lancet* (*N* = 35; 1.9%), *BMJ Open* (*N* = 34; 1.9%), *Antimicrobial Agents and Chemotherapy* (*N* = 24; 1.3%), and *Journal of Infectious Diseases* (*N* = 23; 1.3%) (Supplemental Fig. 4).

## Discussion

This bibliometric analysis systematically evaluated the scientific outputs associated with the WHO/TDR CRDF programme over 22 years, generating evidence on research productivity, authorship progression, collaboration networks, and thematic engagement with infectious diseases of poverty among the fellows. We have to admit that this analysis is limited by the nature of bibliometric data itself. These metrics often fail to show how research actually influences policy, how it’s put into practice, or whether it truly promotes equity which is the whole point of strengthening research capacity strengthening [13]. Our findings do show a link between these fellowships and increased scientific productivity gauged by the publication volumes, better international networking, and more senior roles on the publications. We believe that the fellowships likely contributed to these shifts, but we need to monitor the sustainability of such progress.

On a concrete level, CRDF fellows produced a significant amount of peer-reviewed work, most of which was published after they finished their programs. This temporal pattern appears consistent with previous evaluations of research training initiatives that have reported increased publication activity following structured research training and international exposure. We see a similar trend mirrored in bibliometric evaluations of programs like the National Institutes of Health Fogarty International Center [32] and African research consortia [17]. Across the board, publication output generally increases once a researcher completes a fellowship or grant, and the observed increased output likely exceeded what would be expected solely based on the underlying secular trend. This trend may suggest that the fellowship contributed to strengthening research capabilities across a broad spectrum, potentially encompassing both clinical trials and health systems research [33].

The distribution of publications across a wide range of research areas and study designs including clinical trials, clinical research, health systems research, and basic science reflects the breadth of skills emphasized during the CRDF placements. Yet, the presence of diverse study designs randomized controlled trials, cohort studies, modelling studies, and evidence syntheses raises questions about whether this diversity represents genuine methodological versatility or merely reflects participation in externally driven research agendas. While publication volume alone does not capture research quality or societal impact, it remains a commonly used indicator of scientific productivity and is relevant in the context of research capacity strengthening in LMICs [10, 33].

Authorship position, employed here as a proxy indicator for research leadership and academic seniority, revealed an apparent shift from predominantly first-author publications pre-fellowship toward increased middle- and last-author positions post-fellowship. This pattern might ostensibly suggest career progression consistent with transitions observed in longitudinal studies of research trainees [34].

In the context of international collaborative research, the predominance of middle authorship reflects participation in large, multi-institutional studies, particularly within North–South collaborations. While the proportion of last-author publications remained smaller than first- or middle-author roles, its increase after the fellowship suggests growing involvement in senior research roles. These findings align with the fellowship’s design, which emphasizes both technical research skills and leadership competencies, and support the relevance of authorship patterns as one indicator of evolving professional roles [35].

Collaboration patterns further illuminate that North–South partnerships accounted for the majority of collaborative outputs (Fig. 4A). This pattern shows how institutions in high-income countries often serve as hubs for large multicenter studies. The increase in North–South collaborative publications after fellowship completion is consistent with the CRDF’s objective of integrating LMIC researchers into international research networks.

Although South–South and national collaborations accounted for a smaller proportion of publications, their presence indicates engagement beyond exclusively North-led partnerships. Notably, telemedicine and other digital platforms may further support South–South collaboration by facilitating peer-to-peer exchange, regional clinical trial coordination, and shared learning across LMIC institutions in a cost-efficient manner [36]. Technological innovation increasingly bridges research, education, and clinical implementation, reinforcing the institutional impact of research capacity strengthening initiatives [37]. Previous studies have emphasized the importance of South–South collaboration for strengthening regional research ecosystems and fostering contextually relevant research agenda [38–40]. While bibliometric data alone cannot capture the quality or equity of these collaborations, the observed patterns provide a descriptive overview of the networks in which CRDF fellows participated. While this trend should be monitored over a longer period, it suggests that decades of international investment by various stakeholders are beginning to bear fruit. However, as emphasized in the Abuja Declaration, national investment is essential for building a sustainable R&D ecosystem in LMICs [41].

Several publication characteristics observed in this study are relevant to institutional capacity strengthening. First, the substantial proportion of publications focused on health systems and clinical research suggests engagement with locally relevant research questions that extend beyond disease-specific investigations. In LMICs health systems research has been increasingly taken as a critical component of building sustainable research capacity.

Second, the thematic distribution of publications across infectious diseases of poverty, emerging global health priorities (such as antimicrobial resistance and COVID-19), and non-communicable diseases reflects temporal alignment with evolving health challenges in LMICs. This array of research topics indicates that the scholarly pursuits of the fellows were not restricted to a limited range of subjects but rather encompassed various priority domains pertinent to both national and global health initiatives. Furthermore, the substantial percentage of publications in open-access journals facilitates the widespread distribution of research outcomes, a critical factor for enhancing institutional visibility and promoting knowledge exchange in resource-constrained environments. The significance of open-access publishing is significant for institutions in LMICs, where accessing scientific literature is difficult due to high subscription cost. Collectively, these attributes of publication serve as indirect evidence of contributions that are pertinent to the research landscapes of institutions, encompassing the diversification of research agendas, engagement with health systems inquiries, and dissemination practices that adhere to the principles of global accessibility [10, 12].

### Limitations of the study

This analysis did not include outputs from 22 alumni who did not provide ORCID identifiers, limiting completeness. It focused on peer-reviewed publications linked to ORCID, which likely excludes contributions through policy briefs, reports, and conferences. Bibliometric indicators such as journal impact factor may not reflect public health impact or equity considerations, given structural barriers for LMIC authors and limited open-access options.

Some journals, particularly regional or non-indexed journals from LMIC settings, were not indexed in SCIE/Web of Science or SCImago databases, limiting the availability of standardized journal metrics for a subset of publications.

This study classified publications as pre- or post-fellowship based on publication year. Because research projects often span extended timelines, some post-fellowship publications may have been initiated prior to the fellowship, and conversely, some pre-fellowship publications may have benefited from mentorship or revisions occurring during the fellowship. Therefore, findings should not be interpreted as direct causal attribution of individual publications to fellowship participation but rather as reflecting temporal patterns consistent with contribution-based evaluation.

ORCID registration was not mandatory for all fellows, particularly during earlier programme years. Although ORCID improves author-level disambiguation, incomplete ORCID profiles may have resulted in under-ascertainment of publications for some fellows. Supplementary searches were conducted to minimize this bias; however, some outputs may not have been captured. Additionally, database indexing limitations may affect coverage of regionally published journals. Therefore, results should be interpreted as reflecting documented peer-reviewed outputs rather than the entirety of scholarly contributions. The actual volume of scientific publications reported in this review therefore represent a lower-bound. Finally, Journal Citation Indicator data were unavailable before 2017, requiring imputation that may introduce minor imprecision.

### Strengths of the study

This bibliometric analysis offers a thorough and methodologically sound assessment of the CRDF programme spanning a duration of 22 years. Among the fellows included in the analysis, 24 (31.6%) were from low-income countries, 42 (55.3%) from LMICs, and 10 (13.2%) from upper-middle-income countries. By utilizing ORCID identifiers alongside various journal-level metrics, the investigation meticulously catalogs research output, authorship trends, and collaborative networks among the fellows. It delineates patterns in publication frequency, journal attributes, and the dissemination of open-access materials that occur post-fellowship participation, while also mapping international research collaborations and equity-related aspects of authorship.

The incorporation of a wide range of research domains, methodological approaches, and disease categories significantly bolsters the robustness and applicability of the findings, thereby aligning with global initiatives aimed at combating poverty-associated infectious diseases. Notably, the analysis highlights the growing volume of clinical trial–related publications conducted in LMICs, particularly in sub-Saharan Africa, alongside strengthened institutional and cross-regional networking. These strengths underscore the value of bibliometric approaches for assessing long-term scientific outputs and equity considerations associated with research capacity-strengthening initiatives, offering evidence to inform policy and investment in global health research. Overall, the evaluation of scientific outputs and assessment of bibliometric trends has allowed us to indirectly gauge the long-term outcome of the fellowship for the participant of programme, such as career progression through authorship seniority, expanding network of collaboration, and also increased research capacity in the fellows’ country of origin; all of these remain a particular strength of our evaluation.

### Next steps and future directions

The plan includes surveying alumni to track long-term career and leadership outcomes, analyzing institutional collaboration patterns especially Global South and North–South partnerships and studying shifts in authorship roles post-fellowship to understand evolving mentorship and leadership, using mixed-methods to inform strategies for sustainable research capacity.

## Conclusions

This bibliometric analysis is the first systematic, contribution-oriented assessment of the scientific outputs associated with the CRDF programme between 1999 and 2021. The findings demonstrate sustained research productivity, evolving authorship roles, diverse collaboration patterns, and thematic alignment with global health priorities among the fellows, consistent with the programme’s objectives of strengthening clinical research capacity and leadership. These patterns indicate that the CRDF programme has contributed to research capacity development at both individual and collaborative levels, while generating outputs relevant to institutional research environments in the Global South. These findings demonstrate the value of continued investment in structured clinical research training initiatives while underscoring the importance of integrated evaluation frameworks to understand their full and lasting impact on equitable global health research systems.

## Supplementary Information

Additional file 1.

Additional file 2.

## Data Availability

The data supporting the findings of this study are derived from publicly accessible sources, including ORCID records, PubMed, journal websites, SCImago Journal Rank, Clarivate Journal Citation Reports, and the Directory of Open Access Journals. The curated bibliometric dataset and analytic code are available in GitHub (https://github.com/dawitgetachewa/WHO-TDR-CRDF-Bibliometric).

## Abbreviations

AFR: WHO African Region
AMR: Antimicrobial resistance
AMRO: WHO Region of the Americas
COVID-19: Coronavirus disease 2019
CRDF: Clinical Research and Development Fellowship
DOAJ: Directory of Open Access Journals
DOI: Digital object identifier
EDCTP: European & Developing Countries Clinical Trials Partnership
EVD: Ebola virus disease
GNI: Gross National Income
GSK: GlaxoSmithKline
H-index: Hirsch index
HIV: Human immunodeficiency virus
HIV/AIDS: Human immunodeficiency virus/acquired immunodeficiency syndrome
ICH: International Council for Harmonisation of Technical Requirements for Pharmaceuticals for Human Use
IF: Impact Factor
IQR: Interquartile range
JCI: Journal Citation Indicator
JCR: Journal Citation Reports
LMICs: Low- and middle-income countries
M&E: Monitoring and evaluation
NCDs: Non-communicable diseases
NTDs: Neglected tropical diseases
ORCID: Open Researcher and Contributor ID
ORCID iD: ORCID identifier
PDPs: Product development partnerships
PK/PD: Pharmacokinetics/pharmacodynamics
R&D: Research and development
RCTs: Randomized controlled trials
SDGs: Sustainable Development Goals
SEAR: WHO South-East Asia Region
SJR: SCImago Journal Rank
STIs: Sexually transmitted infections
TB: Tuberculosis
TDR: Special Programme for Research and Training in Tropical Diseases
UHC: Universal Health Coverage
WHO: World Health Organization
WPR: WHO Western Pacific Region
Q1–Q4: Journal quartiles
