# Supplementary material for: Evaluation of scientific outcomes of TDR-supported clinical research and development fellows in low- and middle-income countries: a bibliometric analysis
Source: Infect Dis Poverty. 2026 Jul 1;15:73. doi: 10.1186/s40249-026-01474-1 (PMC13321981; doi:10.1186/s40249-026-01474-1)
Supplement: Supplementary file 1 — Additional file 1. [file 40249_2026_1474_MOESM1_ESM.docx]

**Supplemental figure 1: Journal metrics by time relative to fellowship year**


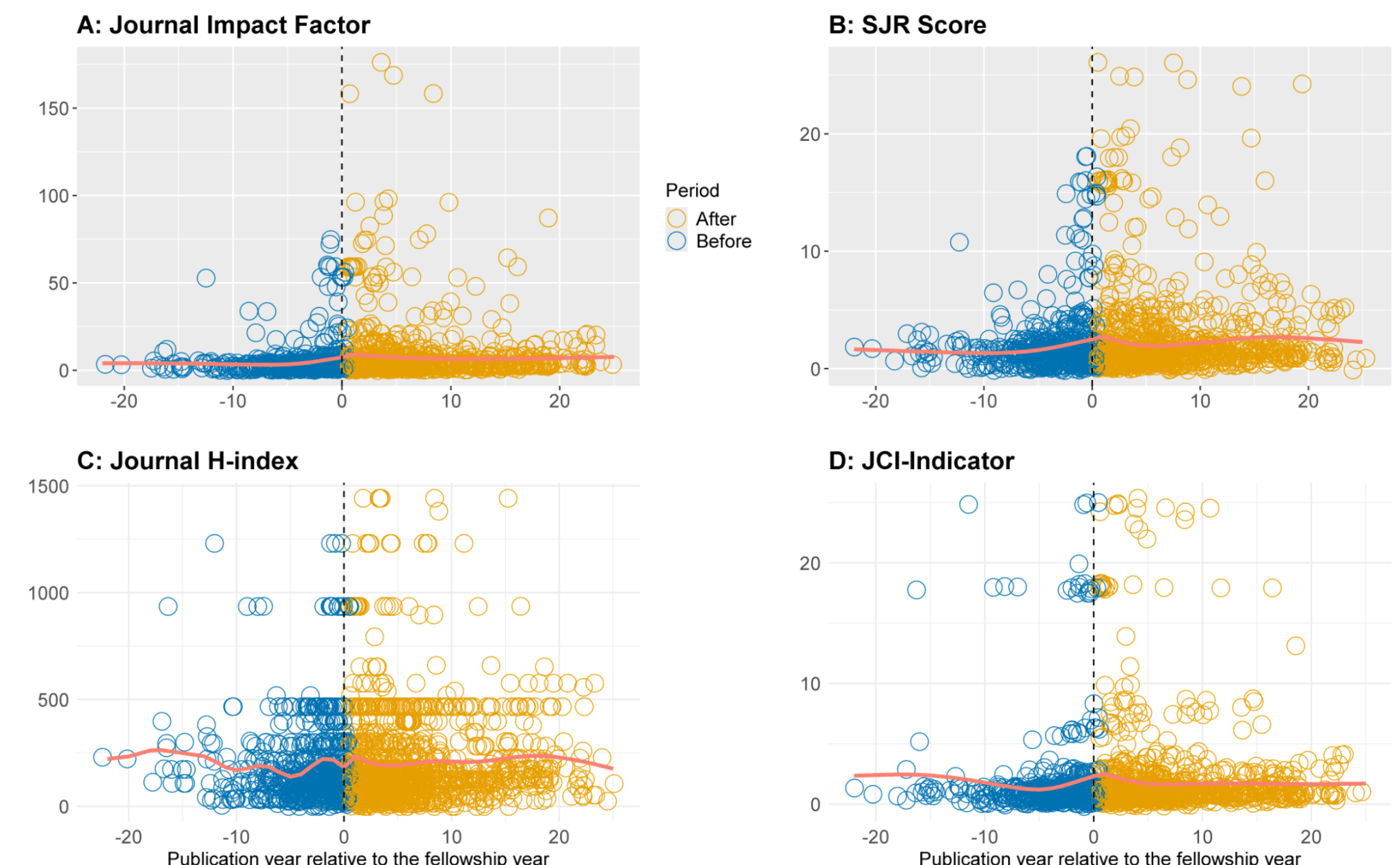


**Legend**: The salmon line represents the period specific trend line derived separately for the before and after period.

**Supplemental figure 2:** Research by disease areas over calendar time


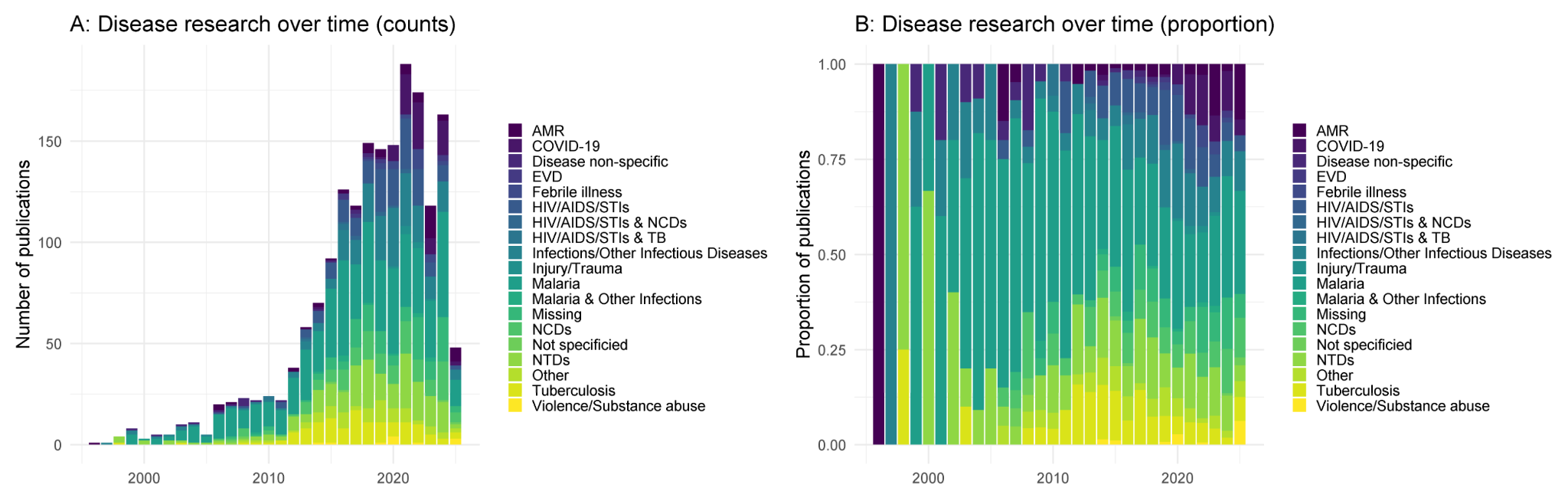


**Legend:** AMR = Antimicrobial resistance; EVD = Ebola Virus Disease; NCDs = Non-communicable diseases; NTDs = Neglected tropical diseases; TB = Tuberculosis; STIs= Sexually transmitted infections.

**Supplemental figure 3**: Breakdown of publication by disease areas, before and after fellowship


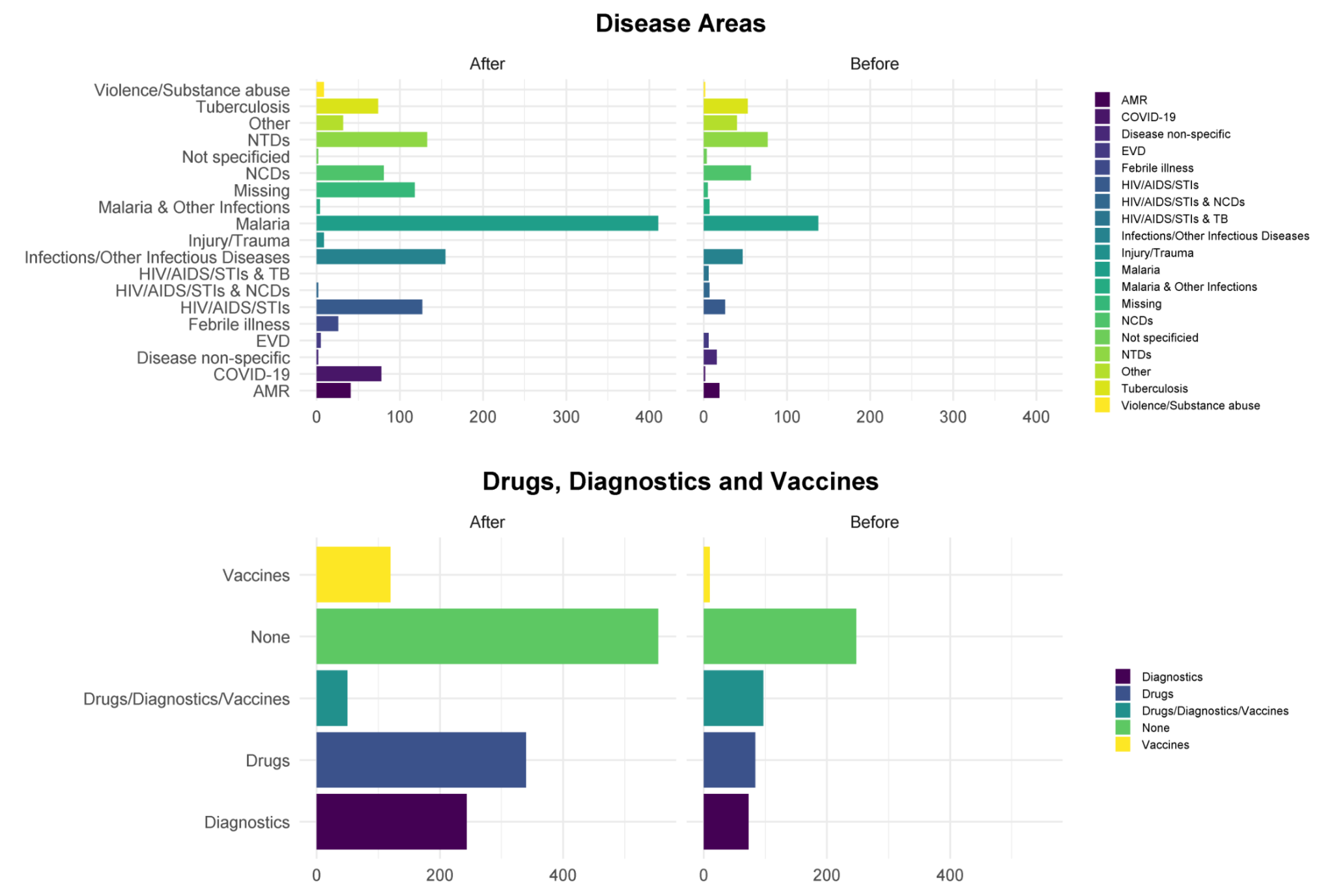


**Legend:** AMR = Antimicrobial resistance; EVD = Ebola Virus Disease; NCDs = Non-communicable diseases; NTDs = Neglected tropical diseases; TB = Tuberculosis; STIs= Sexually transmitted infections.

**Supplemental figure 4: Most frequent journals**


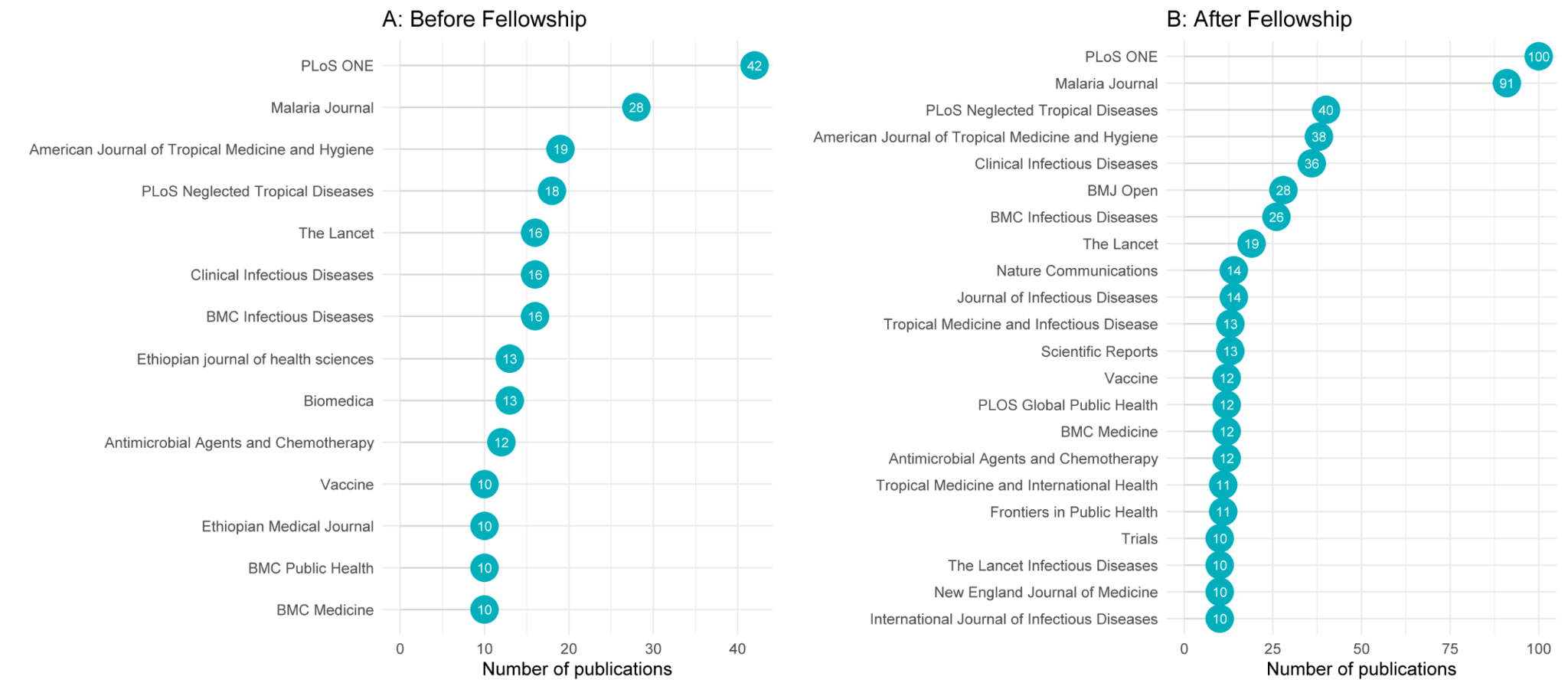


**Legend**: Different range for X-axes across the two panels.
