# Supplementary material for: Evaluation of scientific outcomes of TDR-supported clinical research and development fellows in low- and middle-income countries: a bibliometric analysis
Source: Infect Dis Poverty. 2026 Jul 1;15:73. doi: 10.1186/s40249-026-01474-1 (PMC13321981; doi:10.1186/s40249-026-01474-1)
Supplement: Supplementary file 2 — Additional file 2. [file 40249_2026_1474_MOESM2_ESM.docx]

**Supplemental Table 1: Summary of the WHO/TDR Clinical Research and Development Fellowship Fellows (n=128 fellows)**

|  | **African Region**  **(n=106)** | **Region of the**  **Americas (n=9)** | **Eastern**  **Mediterranean (n=1)** | **South East Asia (n=8)** | **Western Pacific (n=4)** | **Overall**  **(n=128)** |
| --- | --- | --- | --- | --- | --- | --- |
| **Time-Period of fellowship** | 2009–2021 | 2010–2021 | 2016 | 2016–2021 | 2010–2014 | 2009–2021 |
| **Number of countries** | 28 | 4 | 1 | 3 | 3 | 39 |
| **Gender** |  |  |  |  |  |  |
| **Men** | 76 (71.7%) | 5 (55.6%) | 0 (0%) | 6 (75%) | 2 (50%) | 89 (69.5%) |
| **Women** | 30 (28.3%) | 4 (44.4%) | 1 (100%) | 2 (25%) | 2 (50%) | 39 (30.5%) |
| **Number of fellows trained at TPO located in WHO region** |  |  |  |  |  |  |
| **African Region** | 5 (4.7%) | 0 (0%) | 0 (0%) | 0 (0%) | 0 (0%) | 5 (3.9%) |
| **European Region** | 86 (81.1%) | 5 (55.6%) | 1 (100%) | 2 (25%) | 2 (50%) | 96 (75%) |
| **Region of the Americas** | 9 (8.5%) | 4 (44.4%) | 0 (0%) | 1 (12.5%) | 0 (0%) | 14 (10.9%) |
| **Western Pacific Region** | 5 (4.7%) | 0 (0%) | 0 (0%) | 4 (50%) | 1 (25%) | 10 (7.8%) |
| **Eastern Mediterranean** | 0 (0%) | 0 (0%) | 0 (0%) | 0 (0%) | 0 (0%) | 0 (0%) |
| **South East Asia Region** | 0 (0%) | 0 (0%) | 0 (0%) | 0 (0%) | 0 (0%) | 0 (0%) |
| **Multiple** | 1 (0.9%) | 0 (0%) | 0 (0%) | 1 (12.5%) | 1 (25%) | 3 (2.3%) |
| **ORCID ID** |  |  |  |  |  |  |
| **Not available** | 25 (23.6%) | 2 (22.2%) | 0 (0%) | 2 (25%) | 2 (50%) | 31 (24.2%) |
| **Available** | 81 (76.4%) | 7 (77.8%) | 1 (100%) | 6 (75%) | 2 (50%) | 97 (75.8%) |
| **Record of a publication** |  |  |  |  |  |  |
| **No** | 43 (40.6%) | 3 (33.3%) | 1 (100%) | 3 (37.5%) | 2 (50%) | 52 (40.6%) |
| **Yes** | 63 (59.4%) | 6 (66.7%) | 0 (0%) | 5 (62.5%) | 2 (50%) | 76  (59.4%) |
